# Modelling 5-km Running Performance on Level and Hilly Terrains in Recreational Runners

**DOI:** 10.3390/biology11050789

**Published:** 2022-05-22

**Authors:** Onécimo Ubiratã Medina Melo, Marcus Peikriszwili Tartaruga, Edilson Fernando de Borba, Daniel Boullosa, Edson Soares da Silva, Rodrigo Torma Bernardo, Renan Coimbra, Henrique Bianchi Oliveira, Rodrigo Gomes da Rosa, Leonardo Alexandre Peyré-Tartaruga

**Affiliations:** 1 Exercise Research Laboratory, Physical Education, Physiotherapy and Dance Department, Universidade Federal do Rio Grande do Sul, Porto Alegre 90690-200, Brazil; biratri@yahoo.com.br (O.U.M.M.); edsonsoaressilva@hotmail.com (E.S.d.S.); rodrigo_taekwondo@hotmail.com (R.T.B.); renan_coimbra@hotmail.com (R.C.); henriquebianchi30@gmail.com (H.B.O.); rodrigogomesdarosa@gmail.com (R.G.d.R.); 2 Biomechanics Laboratory, Department of Physical Education, Universidade Estadual do Centro Oeste, Guarapuava 85015-430, Brazil; mtartaruga@unicentro.br; 3 Postgraduate Program in Physical Education, Universidade Federal do Paraná, Curitiba 80060-000, Brazil; borba.edi@gmail.com; 4 Postgraduate Program in Movement Sciences, Universidade Federal do Mato Grosso do Sul, Campo Grande 79070-900, Brazil; daniel.boullosa@gmail.com

**Keywords:** anthropometry, athletic performance, cardiorespiratory fitness, endurance training, locomotion

## Abstract

**Simple Summary:**

In the last decades, performance models have helped to comprehend the mechanisms involved in long-term physical performance. In addition, predictive models have aided in the evaluation and prescription of physical training. Here, we tested the hypothesis that physiological assessments under inclined conditions would better explain hilly running performance. We also checked the predictive role of running biomechanical, anthropometric, and neuromuscular factors. Velocity associated with maximal oxygen consumption was more predictive when assessed in inclined conditions (7%) than testing at level. Secondarily, ventilatory thresholds submaximal heart rate improved the performance models at hilly and level conditions. Spatiotemporal, strength, and anthropometric factors were not determinants of performance. Physiological assessments in inclined conditions predict 5-km running performance at hilly terrains in higher degree than evaluations at level in endurance runners.

**Abstract:**

Incline and level running on treadmills have been extensively studied due to their different cardiorespiratory and biomechanical acute responses. However, there are no studies examining the performance determinants of outdoor running on hilly terrains. We aimed to investigate the influence of anthropometrics, muscle strength, and cardiorespiratory and gait spatiotemporal parameters during level (0%) and inclined (+7%) running on performance in level and hilly 5-km races. Twenty male recreational runners completed two 5-km outdoor running tests (0% vs. +7% and −7%), and two submaximal (10 km·h^−1^) and incremental treadmill tests at 0 and 7% slopes, after complete laboratory evaluations. The velocity at maximal oxygen consumption (VO_2_max) evaluated at 7% incline and level treadmill running were the best performance predictors under both hilly (R^2^ = 0.72; *p* < 0.05) and level (R^2^ = 0.85; *p* < 0.01) conditions, respectively. Inclusion of ventilatory and submaximal heart rate data improved the predictive models up to 100%. Conversely, none of the parameters evaluated in one condition contributed to the other condition. The spatiotemporal parameters and the runners’ strength levels were not associated to outdoor performances. These results indicate that the vVO_2_max evaluated at similar slopes in the lab can be used to predict 5-km running performances on both level and hilly terrains.

## 1. Introduction

Performance prediction is an essential part of the athletic training process, as it allows coaches and runners to better design training programs and competitive running strategies. Previous evidence suggests that multifactorial models are useful for identifying the factors that affect distance running performance [1]. From a kinematic point of view, it has been demonstrated that competitive and recreational runners have distinct, ‘typical’ running patterns, as increased knee flexion and decreased ankle eversion at the end of the contact phase found in competitive (faster) runners than in recreational (slower) runners [2], while it has been reported that there is no relation between these parameters and metabolic economy [3]. Furthermore, the running biomechanics is an useful tool to reduce the risk of injuries in lower limbs. A recent study has demonstrated that an increased stride frequency may reduce peak impact force in an outdoor setting [4]. From a physiological point of view, it is well accepted that maximum oxygen consumption (VO_2_max), anaerobic threshold and cost of transport (CoT) can assist in predicting distance running performance [5]. In addition, the so-called neuromuscular factors have also been demonstrated to positively influence distance running races [6,7], showing that the neuromuscular ability to produce maximal muscle force is related to running performance on level terrains [8]. Due to characteristics on positive work production markedly in uphill, the maximal muscle force at slow velocity is expected to be an explanatory factor of performance in hilly terrain. Thus, the best biomechanical, physiological and muscular strength parameters need to be identified to better predict running performance in different conditions of running.

Previously, the velocity associated with the lactate threshold, CoT, and fat-free mass explained 71% of the variance in 5-km running performance in young runners [9]. In another study, the lactate threshold explained 84% of the variance in performance in a 5-km timed trial in veteran runners [10]. Therefore, metabolic and muscular strength parameters seem to be the main predictors of 5-km level-ground running performance. However, it is not clear whether these parameters can explain 5-km running performance under hilly conditions. For instance, inclined running requires a higher metabolic cost by requiring more positive mechanical work [11]. Thus, physiological variables, such as VO_2_, heart rate, and blood lactate concentration, are also higher at the same velocities during inclined running than during level-ground running [7]. It has been shown that an increase in positive inclination by 1% (0.6 degrees) corresponds to a decrease in steady-state velocity by 0.1–0.3 km·h^−1^ [12]. In addition, sloped terrains also promote many biomechanical changes during running [13]. Running on sloped surfaces places specific mechanical demands on the musculoskeletal system since the magnitudes of positive and negative works performed are no longer equal as in level condition. External mechanical work is performed mainly to elevate and lower the body center of mass when moving uphill and downhill, respectively [11]. Although stride time is shorter during uphill running than during level-ground running, the duration for which positive mechanical work is generated is longer due to the longer contact time [14,15]. Therefore, the bouncing mechanism gradually disappears to contain the increase in muscular power (push in uphill vs. brake in downhill) by decreasing the downward (in uphill) versus the upward (in downhill) displacement of the body center of mass. Specifically, uphill running is characterized by a higher step frequency, increased internal mechanical work, shorter swing/aerial phase duration, and greater duty factor in comparison to downhill running. Furthermore, the downhill is characterized by reduced step frequency, decreased duty factor and increased aerial time [15].

The ability to predict performance in hilly distance running may lead to a better understanding of the characteristics of training and improved performance among distance runners. Previous findings have found better relation between CoT and performance when slope-specific analyzed [15]. Furthermore, performance models applied to trial running have shown that VO_2_max and fat mass explained 84% of the performance in short trial running [16]. Moreover, running economy at specific gradients and muscle endurance have been also related to short trial running performance [17]. Although running races for recreational runners are frequently performed in outdoor circuits with different inclined conditions, it is still unknown whether different biomechanical, cardiorespiratory and muscular strength parameters can explain running performance under these conditions. Therefore, the main purpose of this study, was to determine which parameters are the main predictors of 5-km performance in level-ground and hilly running in recreational runners. We hypothesized that outdoor running performance is mostly influenced by parameters assessed in similar and specific conditions (level vs. inclined) in the laboratory, with muscle strength being more important for running hilly races. We choose to represent the hilly conditions at 7% as this incline percentage is commonly used in outdoor races [18] and presenting a difference between positive and negative mechanical work higher than 10% [19].

## 2. Materials and Methods

### 2.1. Study Design

In this cross-sectional study, the participants completed maximal and submaximal treadmill running tests under level (0%) and inclined (7%) conditions, as well as maximal running performance tests on outdoor tracks under level (0%) and hilly (five laps around a one-km street track with 500 m of a +7% uphill slope and 500 m of a −7% downhill slope) conditions. The participants performed five test sessions on different days, with a minimum and maximum interval of three and seven days, respectively, between sessions. In the first session, the participants: (1) signed the consent form, responded to questionnaires, were familiarized with the protocols, underwent the anthropometric data collection, and performed the VO_2_max test under the level condition. The second, third, fourth and fifth sessions were performed in a random order (simple randomizing method in www.randomizer.org (accessed on 3 December 2021)) and consisted of; (2) a VO_2_max test under the inclined (+7%) condition; (3) a 5-km running performance test under the level condition (0%); (4) a 5-km running performance test under the hilly condition; and (5) submaximal running tests (0% and 7%) and a maximal strength evaluation. All participants were made aware of the potential risks, discomforts, and benefits associated with participation before signing the consent form.

### 2.2. Participants

The study sample comprised 20 male recreational runners who trained and competed in local running clubs. The participants were injury and pain-free at the time of the study and had participated in 3-km, 5-km, 10-km, and half-marathon races. They were classified as recreational runners based on their age, sex and 5-km performance times using the USA masters age grading system calculated a posteriori (mean of 58% as a percentage of the world record for the 5-km) [20]. Furthermore, all participants were habituated to run on hilly tracks. The inclusion criteria for the present investigation were to be free from injuries that can affect performance within the last six months, and to have been training regularly (no more than three consecutive days without training) for the last year. The sample size calculation indicated that 15 runners were needed for detecting an association with CoT and the velocity associated with VO_2_max (vVO_2_max) (power = 0.90 and *α* = 0.05, minimal detectable difference of 1.5) [3,21]. Therefore, 20 runners were finally included, considering that some runners may drop out of the study (http://hedwig.mgh.harvard.edu/sample_size/js/js_associative_quant.html (accessed on 10 July 2021)). The participants had a mean of 4.9 ± 3.6 years of running experience, with a mean weekly running volume of 28.9 ± 13.3 km/week, and a training frequency of three sessions/week. Nine runners reported that they regularly performed resistance exercises for at least 16 months.

### 2.3. Procedures

The anthropometric, biomechanical, cardiorespiratory and muscular strength measures are described below. The VO_2_max and submaximal tests were performed on a treadmill (ATL Inbrasport model, Porto Alegre, Brazil) under level and 7% conditions. The 5-km running tests were carried out on a 400 m running track and an inclined street track. Additionally, the runners performed submaximal tests to evaluate the CoT and biomechanical parameters at 10 km·h^−1^ with 0% and 7% slopes. The maximum strength test included a one-repetition maximum test (1RM) utilizing a 45° leg press machine.

### 2.4. Level and Hilly 5-km Running Maximal Tests

The level and hilly 5-km running maximal tests were the performance tests used in the study, all on a rigid surface (asphalt). A 10-min submaximal warm-up run (10 km·h^−1^ at level slope) preceded all the trials. Then, participants were asked to run the 5-km as fast as possible (5-km time trial). An evaluator offered water at 2 and 4 km, and the runners drunk “ad libitum”. Level running test (0%, LEVEL_5km_) consisted of 12.5 laps of 400 m on an official running track. The hilly running test (+7% and −7%, HILLY_5km_) consisted of five laps around a 1-km street track (0.5 km up and 0.5 km down). The ambient temperature of the outdoor tests was between 18 and 22 °C. All angles were transformed to percentage inclines by multiplying the tangent value of the respective degrees by 100.

### 2.5. CoT and Biomechanical Parameters

CoT evaluation consisted of a 5-min warm-up level run at 8 km·h^−1^ followed by 6 min running on a motorized treadmill (ATL Inbrasport model, Porto Alegre, Brazil) at 10 km·h^−1^ with 0% and 7% slopes in randomized order (simple randomizing method in www.randomizer.org (accessed on 10 December 2020)). The minute ventilation, carbon dioxide, VO_2_, and HRCOT were recorded during the final 2 min of the tests. The calibration of the gas analyzer was performed following the manufacturer instructions. The running economy was considered as the CoT in this study; and it was calculated as follows. Firstly, to calculate the CoT, the average VO_2_ during exercise was subtracted by VO_2_ at stand (average from two last minutes from five minutes at stand position), and transformed to energy units (Joules) based on the substrate and the combustion enthalpy of that specific substrate, and finally, divided by the velocity [22,23].

The spatiotemporal running parameters were determined during the CoT tests. Before the test, six reflexive markers were positioned on the right and left feet (ankle, heel, and medial malleoli). The linear positions of markers were filtered through a ‘zero-lag’ second order Butterworth low pass filter with a cut-off frequency detected by a residual analysis on each marker coordinate [24]. The feet movements were registered using a validated movement analysis system (VICON, Oxford Metrics Group, Oxford, UK) composed of six infrared cameras (three cams Bonita, 1.0 Mpixel; three cams T-Series, 1.3 Mpixel) with a sampling frequency of 200 Hz. These markers were selected to identify spatiotemporal running parameters: stride frequency, stride length, stride time, contact time, and aerial time [25]. For calculation of these variables, ten strides from the 4th min at 10 km·h^−1^ with the slopes of 0% and 7% were used, based on a previous study [3] in the Nexus software (VICON, Oxford Metrics Group, Oxford, UK). The choice for just uphill conditions was due to the importance of uphill conditions recently observed in hilly running performance [21]. We used a validated algorithm to detect automatically the gait events during CoT tests [26].

### 2.6. Repetition Maximum Test (1RM)

One repetition maximum (1RM) was assessed in a 45° leg press machine. Participants were given verbal encouragement throughout the protocol, which consisted of 3–5 attempts with 3 min of passive rest between attempts [27]. Firstly, the runners were asked to perform a 5-min warm-up (walking at 6 km·h^−1^) on a motorized treadmill (Inbramed, Porto Alegre, Brazil) and then performed a specific warm-up consisting of 10 repetitions using 100% of their body masses. No static stretching was allowed through the warm-up or between the attempts. Runners performed the first attempt with 200% of their body masses. The test was finished when participants achieved the highest load in one repetition, and this load was recorded for further analyses. The complete dataset used in this study is available at figshare (https://doi.org/10.6084/m9.figshare.14005982.v1 (accessed on 14 July 2021)).

### 2.7. Anthropometric Assessments

The leg length was determined by measuring the distance between the femur greater trochanter and the ground. The skinfold thickness of several sites (subescapular, tricipital, pectoral, axial, suprailiac, abdominal, and thigh) were determined using a calibrated skinfold caliper (Cescorf, Porto Alegre, Brazil). The body density was estimated using the equation by Petroski [28] for men aged between 18 and 61 years using seven skinfolds and the abdomen and forearm perimeters. The percentage of body fat was calculated using Siri’s formula [28]. A professional evaluator with experience in anthropometric assessments performed these measurements.

### 2.8. Maximal Oxygen Consumption Test (VO_2_max)

The runners firstly performed a warm-up, walking on a treadmill (ATL Inbrasport model, Porto Alegre, Brazil) for five minutes at 6 km·h^−1^. The participants underwent the incremental maximal test following the protocols proposed by Paavolainen et al. (2000) [7]. They started to run with an initial velocity of 8 km·h^−1^, with an increase of 1 km·h^−1^ each minute at a fixed incline of 0%. The VO_2_, CO_2_ and tidal volume were measured by indirect calorimetry throughout an automated gas analysis system (VO2000, Medgraphics, St. Paul, MN, USA) [29] in gas mixing chamber mode (10 s). The gas analyzer was calibrated before each testing session following manufacturer instructions. Temperature, atmospheric pressure and humidity in the laboratory were 21 ± 4 °C, 1013 ± 9 mmHg, and 52 ± 2%, respectively, during all submaximal and maximal laboratory tests. The level incremental test ended when the participants communicated to the researchers through visual signs that they needed to stop the test or if the predicted maximal heart rate was reached, or if the respiratory exchange ratio was higher than 1.15. During maximal tests, the runners were verbally encouraged to reach their maximal effort. The VO_2_max protocol for the uphill condition had the initial velocity of 7 km·h^−1^ with an increase of 1 km·h^−1^ each minute at a fixed incline of +7%.

VO_2_max was identified following the criteria described by Howley, Basset, and Welch [30]. The highest average of five VO_2_ subsequent recordings was considered the valid VO_2_max when, at least, one of following criteria was observed: (i) plateau of VO_2_ with concomitant increase in the velocity (all participants attained the true VO_2_max); (ii) estimated maximum heart rate; (iii) respiratory exchange ratio ≥ 1.1; (iv) rating of perceived exertion (RPE) ≥ 17 (very hard) in the Borg’s scale [31].

The first (VT1) and second (VT2) ventilatory thresholds were determined according to previously proposed method [32] by consensus of three independent researchers. The first ventilatory threshold (individual ventilatory threshold) was calculated from the first increase in ventilation-minute with a rapid rise in the ventilatory equivalent of oxygen consumption with no concomitant increase in the ventilatory equivalent of the carbon dioxide production curve. The second ventilatory threshold (respiratory compensation point) was defined as follows: (a) a systematic increase in the ventilatory equivalent of oxygen consumption; (b) a concomitant nonlinear increase in the ventilatory equivalent of carbon dioxide production; and (c) a reduction in the difference in the inspired and end-tidal oxygen pressure [33]. The vVO_2_max was determined as the minimum velocity of the last completed stage, at which VO_2_max was achieved. During VO_2_max testing, if the runner was unable to complete the last stage, the previous velocity summed to the multiplication of the velocity increment by the completed fraction of the last stage, was defined as vVO_2_max [34]. Peak velocity (Vpeak) was defined as the highest velocity (km·h^−1^) sustained for at least 30 s during the test [7].

### 2.9. Statistical Analysis

The Shapiro–Wilk and Levene tests were used to assess the normality and homogeneity of the data, respectively. The data are presented as the mean ± SD. The Pearson product-moment correlation coefficient (r) was used to assess the relationships between performance and prediction parameters. The criteria for including a parameter in the multiple regression models were a *p* < 0.05, a power of 80%, and a tolerance criterion of r > 0.56 [35].

We developed four predictive models based on the combination of cardiorespiratory, biomechanical, muscular strength, and anthropometric parameters to explain running performances on level and hilly terrains. More details are given elsewhere [36]. The cardiorespiratory parameters included VO_2_max, vVO_2_max, velocity peak (V_peak_), first ventilatory threshold (VT_1_), velocity at first ventilatory threshold (vVT_1_), second ventilatory threshold (VT_2_), velocity at second ventilatory threshold (vVT_2_), CoT, and HR during CoT test (HR_COT_). The biomechanical parameters included stride frequency, stride length, contact time, stride time and aerial time. The muscular strength parameters included 1RM and relative strength (i.e., 1RM/body mass). Finally, anthropometric parameters included height, body mass, body mass index, leg length, and body fat percentage.

Multiple linear regression models (stepwise method) were applied to LEVEL_5km_ and HILLY_5km_ using previously described anthropometric, muscular strength, biomechanical and cardiorespiratory parameters. Firstly, we used the biomechanical and cardiorespiratory parameters obtained from level tests to predict the LEVEL_5km_, and biomechanical and cardiorespiratory parameters obtained from incline tests to predict the HILLY_5km_. The assumed linear relations after visual inspection and the normality was assumed after we tested the multivariate normality using the Shapiro–Wilk test. Thereafter, we developed the predictive models with a crossed approach, i.e., using biomechanical and cardiorespiratory parameters obtained from level tests to predict the HILLY_5km_, and biomechanical and cardiorespiratory parameters obtained from incline tests to predict the LEVEL_5km_. Furthermore, we checked the assumption of multicollinearity verifying the tolerance (<0.1) and variance inflation factor (>10) and that no regression model tested had been violated. All statistical procedures were performed using the Statistical Package for Social Science, version 20.0 (IBM, Chicago, IL, USA). The significance level adopted was *α* = 0.05.

The institutional ethics committee (Universidade Federal do Rio Grande do Sul) approval and the corresponding ethical approval code: 33784014.

## 3. Results

The LEVEL_5km_ performance was 21:53 ± 2:33 min:s (75% of vVO_2_max at level, average pace: 4.38 ± 0.51 min/km), and the HILLY_5km_ performance was 25:4 ± 2:34 min:s (83% of vVO_2_max at 7%, average pace: 5.01 ± 0.52 min/km). Both tests were performed between first and second ventilatory threshold). The characteristics of the individuals are presented in Table 1.

Anthropometric and muscular strength data and their associations with LEVEL_5km_ and HILLY_5km_ performance are described in Supplementary File S1. The anthropometric data and lower limb maximum strength levels were not related to running performance under any condition (*p* > 0.05).

**Figure 1:**
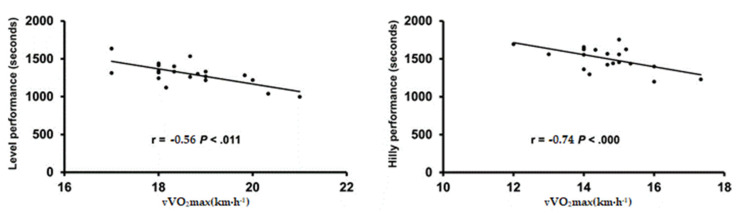
Correlation between level and hilly running performances and velocity associated with VO_2_max (vVO_2_max at level and uphill, respectively).

We found significant correlations between LEVEL_5km_ and HILLY_5km_ and some cardiorespiratory parameters (Figure 1). No significant correlations between running performances and CoT were observed under either condition.

In addition, Table 2 shows that the VO_2_ values at VT_1_ and VT_2_ were similar between conditions. Conversely, CoT was lower under the level condition than under the inclined condition.

Multiple linear regression analyses included only a few cardiorespiratory variables (vVO_2_max, VT_1_, HR_COT_, and VT_2_) for LEVEL_5km_ prediction. The predictive power for LEVEL_5km_ was 80%, with an estimated error of 67.4 s (F = 8.420, 95% CI = −1435.484–4717.419). Similarly, a few cardiorespiratory variables (vVO_2_2max, VT_2_, and HR_COT_) explained HILLY_5km_ performance in the multiple regression analysis. The predictive power for HILLY_5km_ was 69%, with an estimated error of 85.3 s (Table 3, (F = 2.408, 95% CI = −1435.484–4717.419). These multiple regression results indicate that 80% of the variance in LEVEL_5km_ is explained by vVO_2_max in the level test, and 69% of the variance in HILLY_5km_ is explained by vVO_2_max in the inclined test. None of cardiorespiratory parameters evaluated in the level submaximal and maximal tests were able to predict HILLY_5km_. Similarly, none of cardiorespiratory parameters evaluated in incline tests were able to predict LEVEL_5km_. The correlations of the crossed approach are presented in Supplementary File S1.

## 4. Discussion

The objective of this study was to investigate the role of muscular strength, anthropometric, cardiorespiratory and biomechanical parameters for predicting level-ground (0%) and hilly (+7% and −7%) running performances in recreational runners. The primary finding of this study is that the main determinants of running performance under both level and hilly conditions are cardiorespiratory parameters, which does not support our hypothesis that muscle strength is one of the main predictors of running performance under hilly terrain conditions. It is worth noting that specific vVO_2_max plays a critical role in 5-km running performance under both conditions. Importantly, this study examined, for the first time, the determinants of running performance on a hilly terrain, demonstrating that cardiorespiratory rather than muscular strength factors are associated with performance. Furthermore, we tested whether or not evaluations performed at different slopes to those used during races could predict running performances. Our findings show that maximal and submaximal cardiorespiratory variables acquired in level treadmill condition were not predictors of hilly running performance. Likewise, maximal and submaximal cardiorespiratory variables acquired in 7% inclined treadmills were not predictors of level running performance. This finding indicates a hands-on message to coaches, runners and sports scientists reinforcing the need of slope-specific tests for distance runners in line with a previous report on trial running performance [37].

Previous studies have demonstrated that vVO_2_max is associated with level 5-km performance [38]. This result may be attributed to the high VO_2_max percentage demand for this distance. Athletes run 5-km races at approximately 85–95% of VO_2_max [39]. In addition, vVO_2_max represents a combination of VO_2_max, and CoT [40]. Therefore, the metabolic economy at intensities closer to race pace may affect the performance at hilly conditions. The current study extends the findings of previous research by providing a general framework of the main factors affecting running on hills, showing that vVO_2_max is a determinant for not only level-ground but also hilly running performance, and that VO_2_max and CoT do not explain performance, even under inclined conditions.

The weak relation between CoT and performance found under the level and hilly conditions may be attributable to the fact that CoT was evaluated at lower velocity than those carried out during the outdoor performance tests, reinforcing the notion that CoT should be evaluated at velocities similar to those observed in competitive races [41,42]. These findings are in line with previous findings showing a weak correlation between CoT and level running performance in heterogeneous groups [43]. Likewise, the low explanatory power for VO_2_max indicates that it does not distinguish performance in hilly conditions, which has previously been observed on level [38] and inclined [37] terrains. The biomechanical model did not show any associations of the parameters studied with LEVEL_5km_ and HILLY_5km_ performances. These results are different from those reported previously [3], with significant associations between 10 km running performance and kinematics assessed at a constant horizontal velocity higher than that used in the present study (15.8 km·h^−1^). Again, the use of a low velocity (10 km·h^−1^) may have precluded the relationship between CoT and performance in our study, even though the runners in the previous study [3] performed the CoT test at 92% of vVT_2_, which is similar to the metabolic intensity used in our study.

While previous studies have shown that at fixed absolute intensities (same running velocity), no single biomechanical parameters or a subset of parameters can predict CoT [8,44], a more recent study showed an association between stride length, semitendinosus and rectus femoris activation, and CoT, particularly when the metabolic intensity was strictly controlled [3]. In the current study, the gait spatiotemporal model was not able to predict running performance. Nonetheless, this result should be interpreted with caution due to the small number of biomechanical variables used and due to the spring-mass model parameters, which seem to be more strongly related to economy and performance than to spatiotemporal parameters in recreational endurance runners [45,46].

The muscular strength model used here did not show any correlations between the parameters assessed and LEVEL_5km_ or HILLY_5km_ performance. Our findings are in line with previous results [9]. We expected that the hilly running test would offer a condition where the decrease in kinetic energy fluctuations and the increase in gravitational potential energy fluctuations [11] would allow strength-trained individuals to exhibit better performance. The present findings do not suggest a positive influence of muscular strength factors on endurance running performance [6,37,47]. However, other tests more strongly related to muscle power characteristics may reveal significant results. In addition, another potential mechanism mediating the role of muscular power and running performance is post-activation performance enhancement (PAPE) [48,49]. Particularly, PAPE should occur after submaximal endurance work is accumulated, thus counteracting the negative consequences of fatigue and possibly enhancing muscular efficiency [23] and pacing [50]. Additional studies are needed to verify the roles of both muscle power and PAPE in endurance running on different terrains.

Interestingly, no associations were found between the anthropometric model and running performance. This result may be attributed to the homogeneous values (height and leg length variables) recorded from this cohort of recreational runners. For example, the stature of 18 of the 20 runners varied by only 9 cm (173–181 cm), which is consistent with the values reported in a previous study [51] that included a very homogeneous group of individuals in terms of height, body mass, sum of the skinfolds, VO_2_max, and performance time. On the one hand, our findings are also in line with those of a previous study, in which no associations were found between anthropometrics and the 5-km performance time [52]. On the other hand, in the study by Dellagrana et al. [9], significant associations between lean mass and the 5-km performance time were found, suggesting that being leaner and slender [53] may lead to better performance. Again, we did not find significant associations between lean mass and 5-km performance time. We suggest that maturational aspects (18 vs. 26 years) and performance level (~18 min vs. ~21 min) may explain these discrepancies between studies, thus warranting additional investigations.

The strengths of this study are: (i) biomechanical, cardiorespiratory, muscular strength and anthropometric variables were included in the same study; (ii) both performance and cardiorespiratory tests were performed under incline and level conditions; and (iii) the findings of classic studies were confirmed [51] and extended to running under hilly conditions.

The study has some limitations that need to be addressed. We just used men, thus, our findings are not applied to women. The velocity used in the CoT test was lower than that at which the athletes ran in the 5-km test. However, we used this fixed submaximal velocity to guarantee a predominance of aerobic metabolism in both tests. Moreover, our performance test on the hilly terrain was designed to include various slopes rather than uphill sections only, which may have reduced the ability of our models to explain specific performances [54]. However, we designed the test in this manner to find the best predictive model for running performance under real conditions. Moreover, methodological aspects should be considered when the results are interpreted, as the temperature during the outdoor tests varied substantially due to seasonal (i.e., autumn and winter in the Southern Hemisphere) weather conditions. However, this variation was random; therefore, we suggest that its influence on the current results is minimal and provides greater ecological validity to our study. While a consensus is lacking on the best training model to improve the running performance under hilly conditions, from a practical point of view, it seems reasonable to suggest training methods aimed to improve the vVO_2_max on terrains of different inclines.

## 5. Conclusions

In conclusion, the findings of the present study highlight the importance of classic cardiorespiratory parameters that can predict hilly running performance. The current study also showed that spatiotemporal, muscular strength and anthropometric factors do not predict either level or hilly running performance, thus, showing the need for more advanced measurement techniques for these parameters. Interestingly, the predictive models were very similar between conditions, with vVO_2_max being the parameter most strongly associated with outdoor running performance. The velocity associated with VO_2_max combines both maximum aerobic power and metabolic economy; therefore, endurance runners may reach higher velocities at a given VO_2_max when attaining a better CoT. In addition, vVO_2_max can be easily estimated [55] or identified [34] in the field without the need for expensive equipment or a complex setup. Our study shows that slope-specific tests are needed to better predict the outdoor running performances.

## Figures and Tables

**Table 1:** Demographic, anthropometric, muscular strength and performance characteristics of participants.

	Mean	SD	Minimum	Maximum
Age (years)	26.1	6.9	19.0	44.0
Body Mass (kg)	73.9	9.5	55.8	97.7
Height (m)	1.75	0.06	1.58	1.81
Body Fat (%)	8.0	2.8	3.8	12.7
BMI (kg·m^−2^)	24.0	2.3	19.8	29.8
LLL (cm)	92.8	2.9	89	98
5-km level performance (min:s)	21:53	2:33	16:39	27:16
5-km hilly performance (min:s)	25:4	2:34	19:59	29:15
Maximal lower limb strength (kg)	231.2	64.7	120	335
Relative strength (kg·body mass^−1^)	3.1	0.7	1.68	4.05

Standard deviation (SD), Body Mass Index (BMI), Lower limb length (LLL).

**Table 2:** Pearson correlation coefficients (r) and *p*-values (*p*) between level running performance with parameters evaluated at level treadmill (0%, 10 km·h^−1^), and between hilly running performance with parameters evaluated at incline treadmill (7%, 10 km·h^−1^).

Variables	Level Performance vs. Level Treadmill Tests				Hilly Performance vs. Incline Treadmill Tests			
	Mean	SD	r	*p*	Mean	SD	r	*p*
Stride Frequency (stride·s^−1^)	1.38	0.062	−0.527	0.017	1.40	0.62	−0.430	0.058
Stride Length (m)	2.01	0.092	0.500	0.025	1.97	0.089	0.410	0.073
Contact Time (s)	0.003	0.022	0.270	0.249	0.309	0.019	0.034	0.886
Aerial Time (s)	0.620	0.022	0.129	0.588	0.047	0.022	0.066	0.783
VO_2_max (mL·kg^−1^·min^−1^)	50	6.4	−0.460	0.041	50.8	6.0	−0.581	0.007
vVO_2_max (km·h^−1^)	18.3	1.1	−0.744	0.000	14.5	0.8	−0.560	0.011
V_peak_ (km·h^−1^)	18.6	1.2	−0.652	0.001	14.8	0.8	0.582	0.007
VT_1_ (mL·kg^−1^·min^−1^)	30.4	7.6	−0.692	0.001	29.0	6.4	−0.147	0.535
vVT_1_ (km·h^−1^)	11.1	2.02	−0.569	0.009	8.2	0.9	−0.131	0.582
HR at VT_1_ (bpm)	145.0	20.0	−0.059	0.806	135.0	19.0	0.091	0.702
VT_2_ (mL·kg^−1^·min^−1^)	45.4	7.1	−0.649	0.002	45.5	6.0	0.769	0.006
vVT_2_ (km·h^−1^)	15.85	1.5	0.359	0.001	12.8	1.0	−0.534	0.015
HR at VT_2_ (bpm)	178.0	13.0	0.336	0.148	175.0	12.0	0.363	0.116
VO_2_ (mL·kg^−1^·min^−1^)	27.36	5.0	−0.002	0.993	42.1	5.6	−0.281	0.231
CoT (J·kg^−1^·m^−1^)	3.20	0.32	/	/	4.68	0.70	/	/
HR_COT_ (bpm)	136	15	0.771	0.001	165	17	0.768	0.000

Maximum oxygen consumption (VO_2_max), Velocity associated with maximal oxygen consumption (vVO_2_max), Peak velocity (V_peak_), First ventilatory threshold (VT_1_), Velocity at first ventilatory threshold (vVT_1_), Second ventilatory threshold (VT_2_), Velocity at second ventilatory threshold (vVT_2_), oxygen consumption during cost of transport test (VO_2_), Cost of transport (CoT), Heart rate during cost of transport test (HR_COT_). / The correlations from CoT were similar to VO_2_.

**Table 3:** Multiple linear regression using cardiorespiratory parameters from level tests for level running performance and using cardiorespiratory parameters from incline tests for hilly running performance (*p* < 0.05).

Variable Entered in Model	Standardized Coefficient (β)	Partial Eta-Squared	Explanatory Power (%)
Level running perfomance			
vVO_2_max	56.83	−0.442	85.50
VT_1_	4.93	−0.162	7.46
HR_COT_	4.60	0.451	6.67
VT_2_	0.046	0.172	0.07
Total			100
Hilly running performance			
vVO_2_max	34.61	−0.329	72.3
VT_2_	7.99	0.143	16.7
HR_COT_	5.27	0.418	11.0
Total			100

Velocity associated with maximal oxygen consumption (vVO_2_max), First ventilatory threshold (VT_1_), Second ventilatory threshold (VT_2_), Heart rate during cost of transport test (HR_COT_).

## Data Availability

Dataset of Modelling 5-km running performance on level and hilly terrains in recreational runners at https://doi.org/10.6084/m9.figshare.14005982.v1 (accessed on 10 December 2021).

## Supplementary Materials

The following supporting information can be downloaded at: https://www.mdpi.com/article/10.3390/biology11050789/s1, Supplementary File S1: Secondary findings.
