# Supplementary material for: Modelling 5-km Running Performance on Level and Hilly Terrains in Recreational Runners
Source: Biology (Basel). 2022 May 22;11(5):789. doi: 10.3390/biology11050789 (PMC9138284; doi:10.3390/biology11050789)
Supplement: Supplementary file 1 [file biology-11-00789-s001.zip › biology-1702072-supplementary.pdf]

Supplemental material S1 - Secondary findings.

Modelling 5-km running performance on level and hilly terrains in recreational runners

SM1.1 - Correlations between 5-km running performance on level and hilly terrains versus anthropometric and neuromuscular variables in recreational runners

Table S1.1 Pearson correlation coefficients (r) and p-values (*P*) between level and hilly running performance *versus* anthropometric and neuromuscular variables.

Variables	Level		Hilly	
	r	*P*	r	*P*
Body Mass (kg)	-.032	.892	-.089	.708
Height (m)	-.088	.711	-.054	.820
Body Fat (%)	-.086	.718	-.028	.907
BMI (kg.m^{-2})	-.029	.972	-.078	.745
LLL (cm)	-.008	.902	-.003	.989
Maximum ST (kg)	-.334	.150	-.180	.448
Relative ST (kg·body mass^{-1})	-.424	.062	-.194	.413

Body Mass Index (BMI), Lower Limb Length (LLL), Strength (ST).

SM1.2 - Correlations between 5-km running performance on level and hilly terrains versus physiological variables in recreational runners. In this section, we have included the non-specific correlations, i.e., between level performance versus physiological variables determined from uphill (7%) tests, and between hilly performance versus physiological

variables determined from level (0%) tests.

Table S1.2 Pearson correlation coefficients (r) and p-values (*P*) between level running performance *versus* physiological variables evaluated in maximal and submaximal tests at 7% incline.

Variables	level running performance	
	r	*P*
VO_2max	-.404	.070
vVO_2max	-.530	.016
VT_1	-.078	.744
vVT_1	-.285	.223
HR at VT_1	.032	.894
VT_2	-.417	.067
vVT_2	-.758	.001
HR at VT_2	.247	.294
RE	-.298	.202
HR_{RE}	.807	.001

Table S1.3 Pearson correlation coefficients (r) and p-values (*P*) between hilly running performance *versus* physiological variables evaluated in maximal and submaximal tests at 0% incline.

Variables	hilly running performance	
	r	*P*

VO_2max	-.332	.153
vVO_2max	-.633	.003
VT_1	-.400	.080
vVT_1	-.416	.068
HR at VT_1	.027	.911
VT_2	-.464	.039
vVT_2	-.636	.003
HR at VT_2	.316	.175
RE	-.274	.243
HR_{RE}	.653	.002
